# An open-label pilot trial of minocycline in children as a treatment for Angelman syndrome

**DOI:** 10.1186/s12883-014-0232-x

**Published:** 2014-12-10

**Authors:** Joseph C Grieco, Stephanie L Ciarlone, Maria Gieron-Korthals, Mike R Schoenberg, Amanda G Smith, Rex M Philpot, Helen S Heussler, Jessica L Banko, Edwin J Weeber

**Affiliations:** Department of Molecular Pharmacology and Physiology, University of South Florida, Morsani College of Medicine, 12901 Bruce B Downs Boulevard, Tampa, FL 33612 USA; Department of Pediatrics, University of South Florida, Morsani College of Medicine, 12901 Bruce B Downs Boulevard, Tampa, FL 33612 USA; Departments of Psychiatry and Behavioral Neurosciences, University of South Florida, Morsani College of Medicine, 12901 Bruce B Downs Boulevard, Tampa, FL 33612 USA; Department of Neurology, University of South Florida, Morsani College of Medicine, 12901 Bruce B Downs Boulevard, Tampa, FL 33612 USA; University of South Florida Health’s Byrd Alzheimer’s Research Institute, 4001 E Fletcher Avenue, Tampa, FL 33613 USA; Mater Research Institute, University of Queensland, St Lucia, QLD 4072 Australia

**Keywords:** Angelman syndrome, Cognitive impairment, Ataxia, Epilepsy, Seizure, Autism

## Abstract

**Background:**

Minocycline, a member of the tetracycline family, has a low risk of adverse effects and an ability to improve behavioral performance in humans with cognitive disruption. We performed a single-arm open-label trial in which 25 children diagnosed with Angelman syndrome (AS) were administered minocycline to assess the safety and tolerability of minocycline in this patient population and determine the drug’s effect on the cognitive and behavioral manifestations of the disorder.

**Methods:**

Participants, age 4-12 years old, were randomly selected from a pool of previously screened children for participation in this study. Each child received 3 milligrams of minocycline per kilogram of body weight per day for 8 weeks. Participants were assessed during 3 study visits: baseline, after 8-weeks of minocycline treatment and after an 8-week wash out period. The primary outcome measure was the Bayley Scales of Infant and Toddler Development 3rd Edition (BSID-III). Secondary outcome measures included the Clinical Global Impressions Scale (CGI), Vineland Adaptive Behavior Scales 2nd Edition (VABS-II), Preschool Language Scale 4th Edition (PLS-IV) and EEG scores. Observations were considered statistically significant if p < 0.05 using ANOVA and partial eta squared (η^2^) was calculated to show effect size. Multiple comparisons testing between time points were carried out using Dunnett’s post hoc testing.

**Results:**

Significant improvement in the mean raw scores of the BSID-III subdomains communication and fine motor ability as well as the subdomains auditory comprehension and total language ability of the PLS-IV when baseline scores were compared to scores after the washout period. Further, improvements were observed in the receptive communication subdomain of the VABS-II after treatment with minocycline. Finally, mean scores of the BSID-III self-direction subdomain and CGI scale score were significantly improved both after minocycline treatment and after the wash out period.

**Conclusion:**

The clinical and neuropsychological measures suggest minocycline was well tolerated and causes improvements in the adaptive behaviors of this sample of children with Angelman syndrome. While the optimal dosage and the effects of long-term use still need to be determined, these findings suggest further investigation into the effect minocycline has on patients with Angelman syndrome is warranted.

**Trial registration:**

NCT01531582 – clinicaltrials.gov

## Background

First described in 1965, children with Angelman syndrome (AS, DOID_1932) present clinically with physical features such as microcephaly and a puppet like gait as wells as profound developmental delays and little vocal communication ability [1–5]. While these patients exhibit a happy demeanor and easily provoked laughter, this syndrome also consists of other manifestations including hyper-excitability, poor motor function, and delays in adaptive behaviors. Furthermore, patients with AS exhibit EEG patterns specific to the syndrome, and when present in the appropriate clinical context, help in diagnosing the syndrome earlier. Finally, 90% of children diagnosed with AS suffer from seizure of various types and severity [3,6–12].

Angelman syndrome is unique in that nearly all cases result from the disruption of a single gene, *UBE3A* [13,14]. Previous research in both the AS mouse model and humans with AS show no gross morphology changes in brain. However, the absence of the protein product, UBE3A, a E3 ubiquitin ligase, results in the accumulation of regulatory proteins, such as arc and ephexin 5 in the postsynaptic density, which is believed to cause abnormal dendritic spine morphology (filopodial) and density in hippocampal pyramidal neurons leading to aberrant synaptic function [15,16]. These alterations in spine morphology and synaptic function in neurons provides an explanation for the severe behavioral and cognitive manifestations of the syndrome. Our laboratory has recently reported the application of Reelin, a protein shown to increase dendritic spine density, enhanced cognition in a mouse model [17]. Further, other researchers have recently reported the recovery of the cognitive and behavioral deficits associated with AS and even the commencement of UBE3A protein production when certain therapeutics such as UBE3A viral vectors and topoisomerase inhibitors were applied [18–20]. It stands to reason then, a therapeutic with the ability to normalize the aberrant synaptic function underlying AS could ameliorate the severity of symptoms.

Minocycline hydrochloride (MC) is a small (495 kDa), lipophilic, second-generation tetracycline antibiotic medication that readily crosses the blood brain barrier [21]. These characteristics allow minocycline to penetrate the central nervous system more readily than other members of the tetracycline family [22]. As with the aforementioned therapies, minocycline has been shown to recover synaptic dysfunction through the modulation of dendritic spine structure by reducing the activity of matrix metalloproteinases [23]. Previous research has shown the incubation of neuronal cultures with MMP-9 altered dendritic spine shape and number. Moreover, increases in both protein level and activity of the MMP’s occurs in models of epilepsy, which is prevalent in the AS population [12]. Further, minocycline changes the morphology of dendritic spines in hippocampal neurons from elongated (immature) to mushroom-shaped (mature), ultimately rescuing the synaptic defect and improving spatial memory [23].

Interestingly the application of minocycline has been shown to act on numerous other aspects of the CNS. The drug has been shown to be neuroprotective, anti-apoptotic, and anti-inflammatory [24–26]. Beyond this, minocycline can positively alter the AMPA-type glutamate receptor [27–29], metabotropic glutamate receptor 1 and 5 [30,31] and NMDA receptor function [30,32]. Metabotropic glutamate receptors, AMPA receptors and NMDA receptors are known to be important in overall neuronal function and contribute to the synaptic plasticity defect in the AS mouse model [31,33–35].

Minocycline has also been used as a treatment of other human cognitive disorders. For example, when MC was administered to patients with Fragile X syndrome (FRX), significant behavioral improvement in the subscale scores of the Aberrant Behavior Checklist-Community, as well as the Visual Analog Scale and Clinical Global Impressions Scale scores were reported with only minor adverse effects observed [36]. Studies of the drug’s effect on degenerative neuropathology (e.g., Alzheimer’s and Parkinson’s disease, Amyotrophic Lateral Sclerosis) have shown the administration of minocycline reduces the severity and progression of disease and, in some cases, prolongs the lifespan of animal models [37,38].

**Figure 1:**
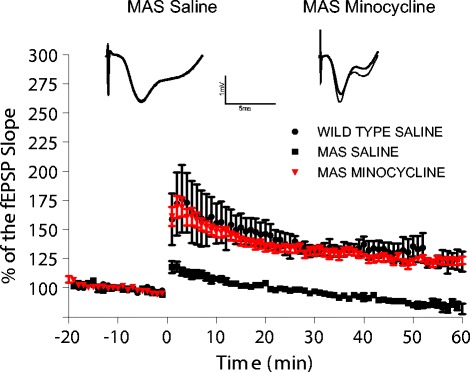
**Minocycline restores the synaptic plasticity defect in the AS mouse model.** 3-month-old UBE3A maternal deficient (AS) mice show increase in long-term potentiation (LTP) after 21 days of treatment with minocycline. Field extracellular postsynaptic potentials (fEPSPs) were recorded and their slopes are conveyed as a percentage of the pre-theta burst stimulation (TBS) baseline. Representative traces before (bold) and 30 minutes after TBS are shown for saline treated (control) and minocycline treated AS mice.

Preclinical electrophysiological studies were carried out in a mouse model of AS (RRID:IMSR_JAX:004477) after 21 days of minocycline treatment. We found a full recovery of the synaptic plasticity defect normally observed in the AS mouse model (Figure 1) [20]. We, and others, have shown a reduction in synaptic plasticity in the hippocampus, cerebellum and visual cortex in the AS mice [34,35,39]. Therefore, recovery of the synaptic plasticity defect was a significant finding for this therapeutic.

The precise mechanism of minocycline, beyond its antibacterial mechanism, is unknown. However, this has not precluded the investigation of minocycline (with associated benefit) on human neurological diseases such as Alzheimer’s, Parkinson’s, Stroke, traumatic brain injuries and Fragile X syndrome [36–38]. The results of the above mentioned studies led us to posit that administering minocycline to patients with Angelman syndrome may ameliorate the central nervous system symptoms associated with the syndrome and improve behavioral performance. Here, we report the changes in symptom severity, cognition, and adaptive behavior after a sample of 25 children with Angelman syndrome were administered minocycline for 8 weeks.

## Methods

### Study design

This study took place at the University of South Florida and was approved by USF’s Human Research Protection Program’s Institutional Review Board (Pro00004716). The USF IRB issued a waiver of assent; however written informed consent was obtained from both the mother and father of each participant. Since no previous research existed showing how children with AS would respond to minocycline, statistical methods could not be employed to determine sample size. Therefore, human subject protections mandated a single arm open-label study design be implemented with no placebo control. After baseline testing (T1), 25 children with AS were prescribed minocycline for 8 weeks. Concurrent administration of medication necessary for seizure control or other comorbid conditions was allowed. The time course and dosing was determined from previous research in which children with FRX were administered minocycline [36]. The study participants were evaluated again after 8 weeks of treatment (T2) and 8 weeks after minocycline was discontinued (T3). The objective of this study was to evaluate the tolerability of minocycline in children with AS and provide preliminary neuropsychological and electroneurophysiology data.

### Recruitment

Participants of this study were recruited through the websites of Angelman parent organizations and clinicaltrials.gov. Children that met the screening criteria were pooled and participants were selected at random using a computer generated randomization schedule (SAS, Cary, NC). To minimize the chance of screen failure, parents were required to have their child’s primary care provider or neurologist complete a short questionnaire attesting the child met the inclusion criteria and provide an indication of the severity of the child’s disability due to Angelman syndrome.

Inclusion criteria included: 1) A molecularly confirmed diagnosis of Angelman syndrome. 2) Male or female. 3) Age between 4 to 12 years old at the time of recruitment. 4) A CGI-S score of 4 or greater indicating at least moderate severity of symptoms. 5) An acceptable surrogate capable of providing consent on the participant’s behalf.

Exclusion criteria included: 1) A known allergy to minocycline or any tetracycline. 2) No molecular confirmation of the AS diagnosis. 3) Participation in another study in which a drug, vitamin or dietary manipulation was used to treat AS within 6 months preceding enrollment. 4) Severe or uncontrolled seizures or any other medical condition rendering the child medically unstable. 5) A history of cardiovascular, respiratory, liver, kidney or hematologic disease or a history of systemic lupus erythematosus.

### Medical and neuropsychological evaluation

Each participant underwent 3 identical study visits consisting of medical evaluation and neuropsychological examination at baseline, after 8 weeks of treatment with minocycline, and 8 weeks after the drug was discontinued. A board-certified pediatric neurologist completed a detailed history and physical examination, assigned a Clinical Global Impressions – Severity (CGI-S) score and interpreted the results of laboratory testing. Blood screening included a complete blood count (CBC), as well as blood urea nitrogen (BUN), creatinine, alanine amino transferase (ALT), and aspartate aminotransferase (AST) levels. Finally, a 30-minute electroencephalogram completed the medical evaluation. Neuropsychological measures were administered during each study visit. These outcome measures included the Bayley Scales of Infant and Toddler Development 3rd Edition (BSID-III), Vineland Adaptive Behavior Scales, 2nd Edition (Vinland-II), and Preschool Language Scale, 4th Edition (PLS-IV). To ensure compliance with the dosing regimen, caregivers were asked to record the date and time each dose of minocycline was administered.

### Safety and adverse event monitoring

Prior to the initiation of any study procedures both parents (or the legally authorized representative) of participants were required to sign an informed consent document in person. The document detailed all of the study procedures and each of the known side effects of minocycline. During the course of the study, caregivers were asked to report any observed side effects and/or changes in behavior immediately via telephone. After 4 and 8-weeks of minocycline treatment as well as 4 and 8-weeks after the drug was discontinued caregivers were asked to complete online questionnaires to document adherence to the medication regimen and to document any observation the caregiver my have made. To assess the safety of minocycline on multiple organ systems, the aforementioned blood-screening tests were reviewed during each study visit. When an adverse event was reported, the duration, severity, relatedness and treatment status were documented (Table 1).

**Table 1:** **A summary of adverse events**

**Participant**	**Description of symptoms**	**Latency to onset (Days)**
1	Feminine Yeast Infection	24^a^
2	Seizure - Atypical Absence	56^a^
3	Commencement of Menstruation	27^a^
4	Difficulty Standing & Balancing	63
5	Dark Spots on Shins	18^a^
8	Lethargy/Sleepiness	18^a,b^
10	Sleepiness	20^a,b^
11	Diagnosis of Lyme Disease	112
13	Urinary Tract Infection	57
15	Seizure - Tonic-Clonic & Difficulty Ambulating	9^a^
21	Influenza Type A	57
22	Sleepiness and Difficulty Ambulating	39^a,b^
24	Seizure	110^c^
25	Seizure Associated with Fever & Vomiting	113

^a^Adverse event occurred during minocycline treatment.

^b^Participant required a dose adjustment.

^c^Subject withdrew due to adverse event.

### Minocycline dosing

After baseline testing, each subject was prescribed minocycline according to his or her body weight (3 mg/kg/day, not exceeding 200 mg a day). The drug was dispensed in 50 mg caplets to be taken orally twice daily (BID). While the lack of speech made it difficult to discern, 3 participants taking 200 mg per day appeared to suffer from intolerable lethargy and/or dizziness (Table 1). The adverse effects resolved when the dose was reduced to 100 mg daily. The dosages used here are equivalent to those used in clinical practice for children greater than 8 years of age and have been established as tolerable and safe in similar patient populations [40,41].

### Statistical analysis

For each dependent measure, the effect of minocycline treatment was assessed using repeated measures analysis of variance (ANOVA) with P values of less than 0.05 considered significant. Post hoc Dunnett’s tests were performed to isolate significant changes from baseline assessment values. A 2 × 3 mixed factor ANOVA was performed with age (≤9 or >9 years old) as a between groups measure and assessment time as a repeated measure. For all analyses, partial η^2^ (effect size) was calculated according to the guidelines of Cohen (0.01 = small effect, 0.06 = moderate effect, 0.14 = large effect) [42].

### Primary outcome measures

Bayley Scales of Infant and Toddler Development, 3rd Edition was administered consistent to the test manual, under the direct supervision of a board-certified neuropsychologist, and by a single pyschometrician who was blind to the purpose and phase of the study. The BSID-III is a measure of development used to assess the cognitive, language and motor abilities of children ages 1 to 42 months. The BSID-III yields scores for five developmental domains: Adaptive Behavior (self-care and self-direction), Cognitive (attention, memory, sensorimotor, and visual preference), Language (receptive and expressive language functions), Motor (fine and gross motor) and Social-Emotional (using emotional signals for self regulation and communication needs). Internal reliability of the BSID-III is high, ranging from 0.086 to 0.93 in healthy subjects. We chose to administer this test because: 1) it has been shown to be reliable and valid with high correlation coefficients for test-retest reliability in children with other neuropathologies [43]; 2) the BSID-III is a common data element suggested by the National Institutes of Health (NINDS) for clinical research involving children with Epilepsy, stroke and other neurologic disorders [44]; and 3) literature suggests the BSID-III is an appropriate measure to use in children, such as those with AS, that exhibit profound developmental delays [45–47]. Under normal circumstances raw scores are converted to standard scores based on age-matched healthy normative data. Children in this sample exhibited raw scores that were well below age-matched peers performances, which would result in standard scores at the floor of the distribution. Past research using the BSID-II in children with AS reported raw scores. Moreover, reporting raw scores adheres to the STROBE reporting guidelines [48]. Finally, utilizing raw scores may better reflect clinical change in functional ability that could be observed for children with profound neurocognitive deficits (e.g., increase in the number of spoken words, or initial expression of speech) that remains far below expectations for age-matched healthy peers and not reflected in standardized scores. Therefore, this study employed raw scores to provide a quantitative assessment of skills and abilities of the BSID-III domains in our analyses [45,49].

### Secondary outcome measure

The secondary outcome measures include the Vineland Adaptive Behavior Scales 2nd Edition (VABS-II) and the Preschool Language Scale 4th Edition (PLS-IV). The VABS-II is a designated NIH common data element for assessment of adaptive skills across 4 behavioral domains: communication, daily living skills, socialization, motor ability and also assesses maladaptive behaviors. Scores are based on subjective ratings of parent’s/primary care provider's perception of a child’s ability to complete various behaviors/tasks. The VABS-II was designed for special needs children, including those with intellectual disabilities, autistic spectrum disorders and ADHD. The test provides normative data for individuals from birth to age 90 years old. Internal reliability for early childhood, birth through 36 months, is 0.79 to 0.95 and varied from 0.83 to 0.93 for children aged 4 to 5 years old. Inter-rater reliability of two different caregivers for the same individual aged birth to 6 years old were moderate to large, ranging from 0.61 to 0.82. [47,50,51]. The PLS-IV is well-recognized interactive, play-based comprehensive assessment of developmental language for children aged birth to 7 years, 11 months of age. Assessment provides scores for Total Language Ability, Auditory Comprehension and Expressive Communication. Internal reliability of measures are generally high, ranging from 0.80 to 0.97. Both the Vineland-II and the PLS-IV have been used extensively in research evaluating developmental language deficits across a variety of developmental disabilities, including Angelman syndrome [52].

### Clinical assessment

A physical examination and EEG assessment was performed at baseline (T1), after 8 weeks of minocycline treatment (T2) and after an 8-week washout period (T3). At every time point, a board-certified pediatric neurologist utilized the clinical global impressions severity scale to rate the severity of the participant’s condition, where 0 represents no symptoms and 7 the most severe symptoms. This scale provides a quantitative measure of symptom severity that allows the clinician to take into account the participant’s history, symptoms, behavior and how his or her disability impacted daily living before and after treatment [53].

A routine 21-channel EEG study was performed utilizing a standard 10/20 system of electrode placement. 30 to 60 minute EEG recordings in the awake and, whenever possible, asleep states were obtained without sedation. Asleep EEG recordings could only be obtained from 3 participants at various time points. After the conclusion of the study, each EEG recording was de-identified, and placed in random order so that the EEG order and relation to treatment were not known. A scoring system was used to evaluate several aspects of the EEG recordings regardless of whether or not they were a part of AS specific EEG patterns. Points were assigned when a particular characteristic was observed. For example, 1 point was given if an EEG was abnormal overall. Characteristics that would be considered more abnormal were scored accordingly. For instance, when evaluating the EEG background, 1 point was assigned if primarily theta waves (mild slowing, >50%) were observed. When a mixture of theta and delta waves (moderate slowing) were observed, 2 points were assigned. Finally, when primarily delta waves (severe slowing, >50%) were recorded 3 points were assigned. Other EEG characteristics were also examined including occipital rhythm (normal-1, slow-2 and absent-3), rhythmic theta (present <50% of the time-1, present >50% of the time-2), rhythmic delta (present-3) and epileptiform abnormalities (present-1, focal-1, multifocal-1, generalized-1, seizure-2). The points were totaled resulting in a total score, ranging from 0 (most normal) to 24 (most abnormal).

## Results

Study Participants: 11 female and 14 male children, mean age 8.2 years old, were enrolled in the study. Of those enrolled, 21 participants were confirmed to have a maternal deletion (the number of deleted bases was variable) and 4 were positive for a mutation of the *UBE3A* gene. Twenty-four children completed the 16-week open-label study; one participant withdrew at week 16 due to unrelated seizure activity.

### Primary outcome measure

A significant improvement in raw scores of the communication subscale of Bayley-III (Table 2) was observed at T3 when compared to baseline, F(2,46) = 3.72, p < 0.05. Post hoc analysis revealed scores from participants between the ages of 4 and 9 years old were responsible for a 40% increase, while scores from participants between ages 9 and 12 years of age remained unchanged. Moreover, a significant improvement, F(2,46) = 5.011, p < 0.05, of the subscale self-direction was observed at both T2 and T3, compared to T1. While no change was observed in gross motor ability, a significant increase (15%) in the measure of fine motor ability was observed at T3, F(2,46) = 3.28, p < 0.05.

**Table 2:** **Neuropsychological outcome measures**

**Bayley Scales of Infant and Toddler Development, 3rd Edition**								
	**Cognitive**	**Communication**	**Language**		**Motor**		**Self**	
			**Receptive**	**Expressive**	**Gross**	**Fine**	**Care**	**Direction**
T1	26.4 ± 2.48	18.9 ± 1.60	13.4 ± 0.61	10.1 ± 0.76	41.3 ± 1.69	19.4 ± 1.05	37.8 ± 1.81	33.8 ± 2.24
T2	31.7 ± 1.83	22.8 ± 1.69	13.7 ± 0.81	11.8 ± 0.75	40.5 ± 1.60	19.8 ± 1.47	40.6 ± 1.84	*38.1 ± 1.98
T3	30.7 ± 2.09	*23.5 ± 1.88	13.8 ± 0.68	11.2 ± 0.68	42.8 ± 1.12	^†^22.4 ± 1.5	40.0 ± 1.99	*39.5 ± 1.94
η^2^	0.05	0.05	0.002	0.04	0.02	0.03	0.02	0.06
**Vineland Adaptive Behavior Scales, 2nd Edition**								
	**Maladaptive Behavior**		**Communication**		**Motor**		**Daily Living Skills**	
	**Internal**	**External**	**Receptive**	**Expressive**	**Gross**	**Fine**	**Personal**	**Domestic**
T1	6.7 ± 0.44	5.5 ± 0.58	30.3 ± 1.62	41.8 ± 3.02	48.3 ± 3.27	99.0 ± 7.12	53.8 ± 5.61	2.6 ± 0.95
T2	5.5 ± 0.47	4.7 ± 0.57	*36.4 ± 2.42	44.3 ± 3.23	48.9 ± 3.32	101.2 ± 7.5	59.1 ± 5.75	3.8 ± 1.49
T3	5.7 ± 0.52	6.0 ± 1.25	33.9 ± 2.25	43.8 ± 2.58	49.4 ± 3.25	95.4 ± 7.84	56.4 ± 4.95	2.7 ± 0.92
η^2^	0.05	0.02	0.06	0.06	0.001	0.004	0.007	0.01
**Preschool Language Scale, 4th Edition**								
	**Auditory Comprehension**		**Total Language**			**Expressive Communication**		
T1	17.64 ± 0.68		34.20 ± 1.58			16.3 ± 1.14		
T2	18.25 ± 0.58		34.88 ± 1.36			16.6 ± 0.94		
T3	*20.48 ± 0.71		*38.83 ± 1.34			17.5 ± 0.71		
η^2^	0.13		0.08			0.001		

Mean ± Standard Error.

η^2^ (Partial Eta squared) is a measure of effect size. 0.01 suggests a small effect, 0.06 a medium effect and 0.14 a large effect.

*p < 0.05 when the time point is compared to T1 (baseline).

†p < 0.05 when the time point is compared to T2 (after treatment with minocycline).

### Neuropsychological outcomes

A significant improvement in the raw scores of the receptive communication index of the Vineland-II was found between T2 and baseline, F(2,46) = 6.73, p < 0.05. Two domains of the PLS-IV, auditory comprehension and total language ability, were both found to have increased significantly when measures at T3 were compared to baseline, F(2,44) = 6.73, p <0.05 and F(2,44) = 5.84, p <0.05, respectively.

### Clinical outcomes

Blood screening tests showed no clinically significant changes over the 16-week study course and no serious adverse effects related to minocycline treatment were reported (Table 1). In 3 cases, caregivers reported lethargy and/or dizziness that required a dose adjustment and was considered related to the minocycline treatment. All of these participants were receiving 100 mg of minocycline BID. In 2 other cases, caregivers reported difficultly standing and/or walking that resolved after the discontinuation of minocycline. Significant clinical improvement over baseline, for all participants, as measured by the CGI-S score (Table 3), was observed at T2 and T3 [F(2, 46) =13.20, p < 0.05]. Analysis of EEG scores revealed a 4.3% and 10.8% improvement when T2 and T3 observations were compared to baseline but did not reach the level of significance [F(2,46) = 1.494, p > 0.05]. For both measures, calculated partial η^2^ was equal to 0.05, an indication of a moderate effect size.

**Table 3:** **Patient populations & clinical measures**

**Participant**	**Sex**	**Age (Months)**	**Daily dosage (mg)**	**CGI**			**EEG**		
				**T1**	**T2**	**T3**	**T1**	**T2**	**T3**
1	Female	100	200	5	4	5	4	7	4
2	Male	88	100	4	4	4	8	9	6
3	Female	124	200	7	6	7	11	7	12
4	Female	128	200	6	5	5	14	12	11
5	Male	119	100	4	4	3	5	5	3
6	Male	160	200	5	4	5	6	6	6
7	Female	89	100	5	5	5	5	9	5
8	Female	101	200	5	5	5	8	5	9
9	Male	60	100	6	5	6	15	9	12
10	Male	86	200	4	4	4	8	8	6
11	Female	89	100	5	5	5	12	9	8
12	Female	112	200	4	3	3	9	8	5
13	Female	111	100	6	4	4	9	9	6
14	Male	76	200	6	6	6	9	9	8
15	Male	127	200	6	5	6	6	6	4
16	Male	63	100	7	6	6	14	11	16
17	Female	72	100	5	4	5	14	12	12
18	Female	83	100	5	4	3	9	9	9
19	Male	97	100	5	4	4	5	7	11
20	Male	125	200	5	5	5	10	15	3
21	Male	130	200	7	7	7	10	9	10
22	Male	148	200	8	7	7	4	3	8
23	Male	120	200	5	5	5	14	13	13
24	Male	87	100	7	7	-	7	9	-
25	Female	85	100	7	6	7	13	13	9
	*Mean ± Standard Deviation*			5.56 ± 1.12	*4.96 ± 1.10	*5.08 ± 1.25	9.16 ± 3.47	8.76 ± 2.80	8.16 ± 3.47
	*Standard Error of the Mean*			0.224	0.220	0.255	0.694	0.561	0.709
	*η* ^*2*^				0.05			0.05	

η^2^ (Partial Eta squared) is a measure of effect size. 0.01 suggests a small effect, 0.06 a medium effect and 0.14 a large effect.

*Indicates p < 0.05 when the time point is compared to T1 (baseline).

## Discussion

To our knowledge, this is the first prospective trial of minocycline treatment in which safety, tolerability, cognitive function and adaptive behavior were evaluated among children with Angelman syndrome. Statistically significant changes were observed across several subscales with moderate relative effect sizes (Table 2) in both the primary and secondary outcome measures for language, communication and self-direction. When the mean scores for all participants were analyzed, a significant improvement in communication and fine motor ability was observed. Participants also showed a significant improvement in self-direction, the ability to entertain themselves and follow simple directions, after being treated with minocycline. This suggests minocycline may have positive effects on language, fine motor skills and some adaptive behaviors. While raw scores had to be used considering the cognitive and language deficits of these children, the improvements in cognition, language and motor skills were perceived by caregivers as shown by the VABS-II results. These pilot data show minocycline may improve the pervasive cognitive and language deficits of these patients over a 16-week period.

The effects of minocycline were also observed clinically as shown by clinician rated function (CGI-S) scores. No change in the EEG scores was observed. However, this was measured with consistent anti-seizure medication and concurrent treatment of minocycline. Thus, it is unclear whether minocycline has an effect on EEG patterns of AS patients not receiving treatment for seizure. Caregivers reported few adverse effects and no severe adverse events related to minocycline treatment. Language limitations required adverse event reports from caregivers rather than participants themselves. In 3 cases, lethargy and/or dizziness was reported in participants taking 200 mg of minocycline daily. These symptoms subsided after the dosage was reduced, suggesting a smaller dosage may be required to mitigate adverse effects. We show statistically significant results after MC treatment was discontinued (T3) suggesting a lasting treatment effect. However, the optimal treatment course may be more extensive. Therefore, further study is needed to determine the optimal length of treatment and what effect extended exposure to minocycline may have in the AS patient population. In general, these data suggest the short-term use of minocycline at these dosages in children with AS is well tolerated.

Several notable limitations of this pilot study include the behavioral manifestations of the participants, single arm study design, lack of control for practice effect, and small sample size. As expected, the behavioral characteristics of the participants (low tolerance for frustration, limited language skills, articulation patterns that made speech intelligible only to caregivers) interfered with their performance on the neuropsychological instruments. However, these behavioral deficits reflect the severe cognitive and language impairments of these individuals and account for their below age level skills in self-care and social behaviors. Moreover, the preponderance of the testing consists of questionnaires and relies on parent responses that may include bias. Regardless, we chose to employ the same neuropsychological instruments used in previous studies of AS patients. This includes the Bayley Scale of Infant and Toddler Development – 3rd Edition despite the age limit of 42 months.

Future research to explore therapeutic benefits of minocycline in AS should involve studies with a placebo-controlled crossover design to better control for practice and temporal effects. The study design also did not allow for evaluation of practice effects particularly from T1 to T2. Previous research is replete with data indicating practice effects are present in neuropsychological assessments and are often most pronounced between first and second testing periods and decrease from second to third testing periods. It is important to point out that there are no published results studying practice effect in children with Angelman syndrome. Moreover, the calculated η^2^, the results reported here show the associated improvement in neuropsychological measures reflected an actual treatment effect not attributable to practice or error.

## Conclusion

The data reported here show the administration of minocycline to children with Angelman syndrome is safe and well tolerated. Moreover, we show minocycline improved the adaptive behavior of these children suggesting this drug may be an effective treatment of this disorder. It is important to determine the optimal treatment dosage as well as the effects of long-term use in this patient population. Therefore, future controlled studies are recommended before minocycline is used generally as a treatment for Angelman syndrome.
